# Bioavailability of Nutrients from Animal-Derived Foods Versus Other Sources of Foods: A Comprehensive Literature Review

**DOI:** 10.3390/nu18172882

**Published:** 2026-09-03

**Authors:** Mariusz Rudy

**Affiliations:** Department of Agricultural Processing and Commodity Science, Institute of Food Technology and Nutrition, Faculty of Technology and Life Sciences, University of Rzeszów, Zelwerowicza 4, 35-601 Rzeszow, Poland; mrudy@ur.edu.pl; Tel.: +48-(0)-17-785-52-60

**Keywords:** nutrients, protein, iron, zinc, calcium, vitamins, bioavailability, animal-based foods, plant-based foods

## Abstract

Modern nutritional science places increasing emphasis not only on absolute nutrient content, but primarily on bioavailability and interactions with the food matrix. This paper presents a comprehensive comparison of the bioavailability of protein, vitamins (A, D, and B_12_), and key minerals (iron, zinc, and calcium) from animal-source foods (ASF) and plant-based alternatives. Based on the literature indexed in the Web of Science, Scopus, PubMed, and ScienceDirect databases, this review assessed nutrient absorption and matrix effects, including both antinutritional factors and health-promoting compounds. The analysis shows that while ASF generally exhibit higher baseline bioavailability of several essential micronutrients and protein (measured by PDCAAS and DIAAS) due to the absence of phytates and oxalates, plant-based matrices offer distinct physiological benefits, including dietary fiber and phytonutrients. Moreover, the lower bioavailability in plant sources can be significantly improved through food processing techniques (e.g., fermentation, germination, thermal treatment) and strategic dietary combinations. Therefore, adequate nutrient intake depends not only on the food’s origin but also on its preparation methods and the overall dietary pattern. It is also important that the best diet is both nutritionally adequate and sustainable for the individual and the environment.

## 1. Introduction

Ensuring optimal nutritional status of the human population requires precise dietary modeling based on the actual bioavailability of nutrients, not just their raw quantitative content in raw food materials [1,2]. Traditional research approaches, focusing solely on simplified, quantitative chemical analysis of food composition and the creation of static tables of nutritional values, are gradually giving way to advanced dynamic models. These models take into account the multi-stage metabolism of a given substance, which includes its bioaccessibility (the degree of release of the nutrient from the food matrix in the intestinal lumen), bioavailability (the efficiency of passage through the enterocyte barrier into the portal or lymphatic circulation), and final metabolic utilization by target tissues [1,3,4]. Ignoring these physiological relationships can lead to serious errors in estimating the nutritional value of diets. For example, the bioavailability of 100 mg of calcium from raw spinach differs from that of cow’s milk due to the different degree of ionization of this element and the presence of organic ligands that limit its absorption [5,6].

This research direction also aligns with current trends toward a radical reduction in the consumption of animal products for ecological, climatic, and ethical reasons [2]. A thorough understanding of the differences in the kinetics of nutrient absorption between plant-based diets and traditional models is crucial [7,8,9]. The human digestive system, characterized by a relatively short large intestine and the dominant role of the small intestine, is highly efficient in digesting and absorbing foods with high nutritional density and lower ballast content, a typical example of which are products of animal origin [8,10]. Databases such as Web of Science, Scopus, PubMed, and ScienceDirect record a significant increase in clinical trials, meta-analyses, and in vitro modeling studies demonstrating that animal-source foods (ASF) can provide a concentrated, complete, and highly bioavailable source of many essential micronutrients. These studies indicate that hasty elimination of ASF without thorough technological adjustments to other dietary components can lead to subclinical systemic deficiencies [11]. The journal Nutrients makes a particular contribution to the precise mapping of these mechanisms, regularly publishing groundbreaking papers on the physiology of membrane transport, cation competition for intestinal transporters, and the impact of innovative food processing methods on the release of mineral compounds from plant complexes [6,8,12].

This paper aims to provide a comprehensive, comparative, and multifaceted overview of the bioavailability of key nutrients (proteins, iron, zinc, calcium, and vitamins A, D, and B_12_) from animal-derived foods and alternative food sources. Particular emphasis is placed on the molecular and biochemical determinants of the physiological barriers of the gastrointestinal tract, interactions occurring within the food matrix, and technological strategies to optimize plant-based diets by reducing antinutritional substances and counteracting the phenomenon of “hidden hunger” (chronic deficiency of key micronutrients: vitamins and minerals, which occurs despite the body receiving sufficient, and often excessive, energy) in society [11].

## 2. Review Methodology

### 2.1. Search Strategy and Data Sources

A literature search was conducted in December 2025 and updated in May 2026 (to identify recently published evidence) using key international databases indexing peer-reviewed publications: Web of Science, Scopus, PubMed, and ScienceDirect. To identify relevant research papers, the following Boolean search query was formulated:

(bioavailability OR bioaccessibility OR absorption) AND (meat OR dairy OR “animal-source foods” OR plant-based) AND (protein OR iron OR zinc OR calcium OR “vitamin B_12_” OR cobalamin OR “vitamin A” OR carotenoids OR “omega-3” OR DIAAS) AND (“clinical trial” OR “in vitro digestion” OR cohort).

The search was limited to papers published in English. The search process was supported by a manual review of the reference lists of the most important review articles to identify additional records. Narrative reviews were included selectively to provide contextual background and mechanistic insights where appropriate.

### 2.2. Study Inclusion and Exclusion Criteria

**Figure 1:**
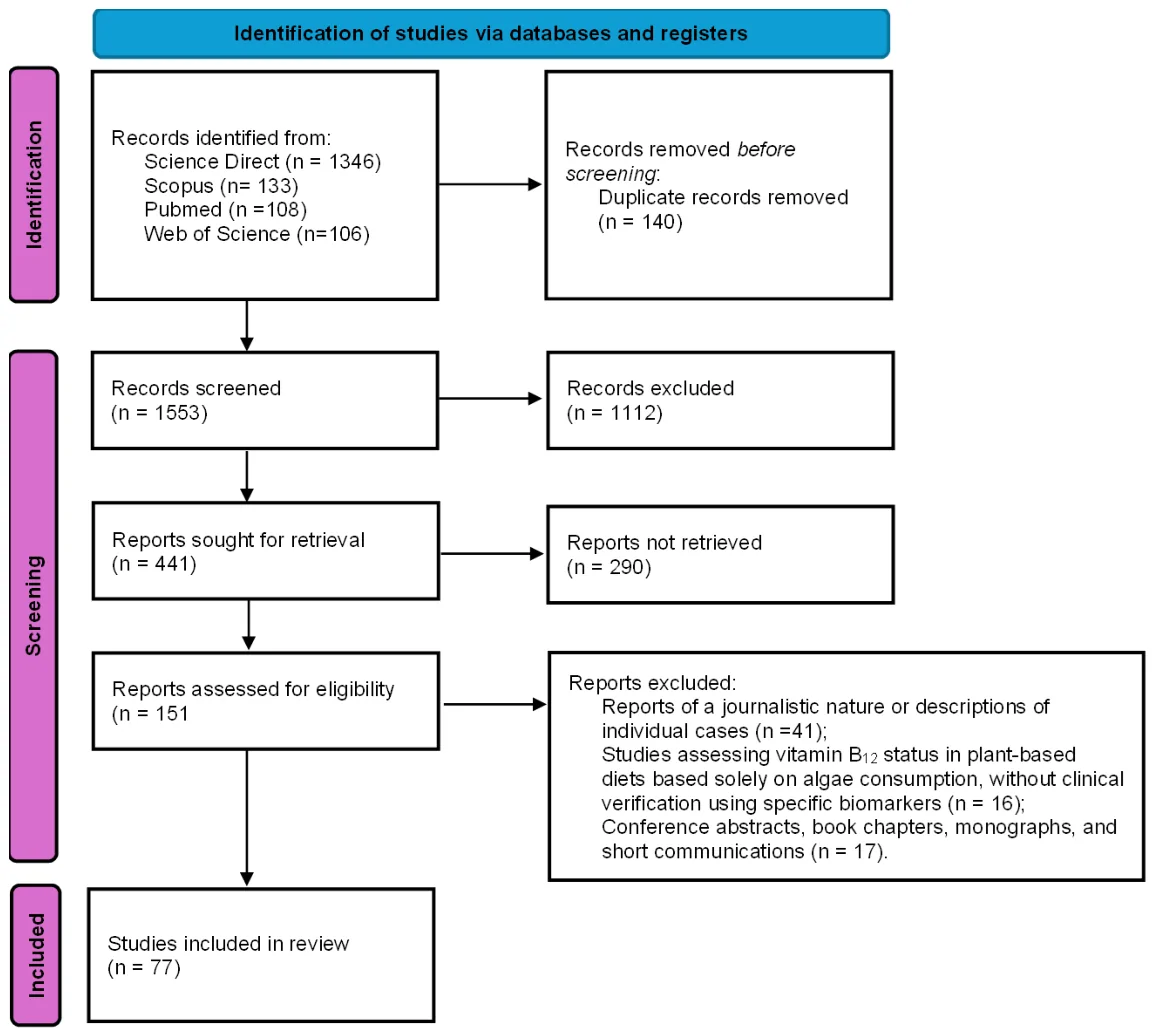
PRISMA flowchart of study selection process [13].

The selection process is presented in Figure 1, which contains a flowchart. The process of selecting identified records was multi-stage:

1. Aggregation and Duplicate Removal: All identified records were exported to bibliographic formats (RIS/CSV) and imported into bibliography management software. Zotero Bibliography Manager 9.0.5 was used to organize and cite scholarly sources. Duplicates were eliminated by comparing unique DOI (Digital Object Identifier) numbers across databases and text normalization of article titles (removing punctuation, converting to lowercase, and eliminating excess spaces).
2. Initial Screening: Based on the titles and abstracts, the review authors independently assessed the articles’ compliance with the inclusion criteria. Any discrepancies were resolved by consensus.
3. Full-text analysis: Articles qualified for the next stage were subjected to a detailed full-text analysis in terms of their methodological completeness and substantive contribution to the achievement of the objectives of this study.

In the process of verifying the identified publications, rigorous selection criteria were applied:

Inclusion criteria:

1. Controlled clinical trials assessing the absorption or plasma concentrations of biomarkers of test nutrients in humans.
2. Studies on animal models carried out to calculate the intestinal digestibility indexes (DIAAS) of dietary proteins [14].
3. In vitro experimental work using recognized physiological digestion standards (e.g., INFOGEST protocol) and Caco-2 enterocyte cell cultures [4].
4. Studies evaluating the physicochemical parameters of the food matrix and the kinetics of component release.

Exclusion criteria:

1. Reports of a journalistic nature or descriptions of individual cases.
2. Studies assessing vitamin B_12_ status in plant-based diets based solely on algae consumption, without clinical verification using specific biomarkers [15].

### 2.3. Data Extraction and Methodological Quality Assessment

Standardized quantitative data (including mean net mineral absorption percentages, DIAAS and PDCAAS values for individual sources, carotenoid-to-retinol conversion factors (RAE), plasma and tissue concentrations) were extracted from the eligible studies and used to create comparative summaries. The methodological validity of the included clinical studies was assessed using validated risk of bias assessment tools [9]. The methodological validity and risk of bias of the clinical trials included in the review were assessed using guidelines and the standard Cochrane Risk of Bias 2 (RoB 2) tool. Assessments were performed independently by two reviewers across five mandatory methodological domains: (1) risk of bias resulting from randomization, (2) risk of bias due to deviations from the planned intervention, (3) risk of bias resulting from missing outcome data, (4) risk of bias in outcome measurement, and (5) risk of bias in reporting outcomes. Based on responses to detailed guiding questions within each domain, individual studies were classified into one of three overall risk of bias categories: “low risk of bias,” “some uncertainty,” or “high risk of bias.” Any discrepancies in assessments between reviewers were resolved through discussion and consensus. The obtained results were considered during quantitative data synthesis using sensitivity analysis. It consisted in excluding studies with a high risk of bias from the combined estimation, which allowed for verification of the impact of the methodological quality of the original reports on the stability and reliability of the final conclusions.

## 3. Comparison of the Bioavailability of Selected Nutrients from Foods of Animal and Plant Origin

Understanding the fundamental differences in the rate of nutrient absorption from different food matrices requires a systematic analysis of the chemical species and the kinetics of their passage through the intestinal barrier. Although plant foods may have high absolute content of many elements and vitamins, their actual metabolic utilization (bioavailability) is determined by the evolutionary structure of the organic matrix and the co-presentation of cofactors or inhibitors [1,8].

Table 1 presents a comparison of chemical forms, primary sources, and average bioavailability coefficients of essential nutrients in humans.

Table 2 presents summary of bioavailability parameters of selected nutrients in clinical studies.

This disproportion results from the collision of two biologically different systems:

Animal matrix (ASF): The ingredients are highly soluble, chelated by specific carrier peptides and released under the mild enzymatic conditions of the gastrointestinal tract [6].

Plant matrix: Performing structural and defensive functions. Plants synthesize a wide spectrum of organic ligands (phytates, oxalates), whose biological role is to bind cations valuable to the plant and prevent their loss [8,15].

Therefore, dynamic dietary modeling should assume that replacing a gram of protein or a milligram of an animal mineral with its plant equivalent without prior technological modification may be physiologically insufficient, potentially resulting in subclinical nutrient deficits [2,9,11]. Although diets rich in ASF are highly nutritious, well-planned plant-based diets can be suitable for most people.

## 4. Protein Quality and Bioavailability Assessment (PDCAAS vs. DIAAS)

Animal proteins found in foods such as meat, poultry, fish, eggs, and dairy products typically provide all essential amino acids in proportions appropriate for human needs [21,22,23]. However, most plant proteins are deficient in one or more essential amino acids, which means that they may be inferior to animal proteins in this respect [21]. A solution to this deficiency may be combining different plant sources in the diet, such as rice and beans, wheat and legumes, or so-called pseudocereals (quinoa, amaranth, buckwheat), to obtain a complete amino acid profile [24,25,26]. Furthermore, plant proteins often contain antinutritional components, such as fiber, phytates, tannins, and protease inhibitors, compounds that can hinder protein digestion and absorption and bind to essential minerals, reducing their bioavailability [27,28,29,30,31,32]. The bioavailability and digestibility of plant proteins are also influenced by factors such as the structure of the protein matrix, cell wall integrity, and protein secondary structure [33,34]. Processing techniques such as milling, alkaline extraction, and isoelectric precipitation also play a key role in improving the digestibility of plant proteins [35,36,37,38,39,40,41]. Interactions amongprocessing, the food matrix, and mechanical degradation significantly impact play protein digestibility [39,42,43,44,45]. Additionally, methods such as cooking, fermentation, germination, extrusion, and the conversion of plant proteins into isolates or concentrates have been shown to be effective in improving digestibility, nutrient profiles, and functional properties [46,47,48,49,50,51,52,53,54,55,56]. By degrading antinutritional compounds and modifying protein structure, these processes typically increase the bioavailability of plant proteins, making them more beneficial and nutritionally valuableto humans.

For decades, the standard recommended by FAO/WHO was the PDCAAS (*Protein Digestibility-Corrected Amino Acid Score*), calculated based on apparent fecal digestibility. This method assumed that the difference between the amount of nitrogen consumed and excreted in the feces reflected the amount of nitrogen absorbed. However, this assumption is fundamentally flawed. The microbiotainhabiting the large intestine can deaminate undigested proteins arriving from the small intestine, using the released nitrogen to synthesize its own structural proteins. As a result, this nitrogen does not appear in the feces in its original form, leading to a false overestimation of the quality of plant sources [10].

**Figure 2:**
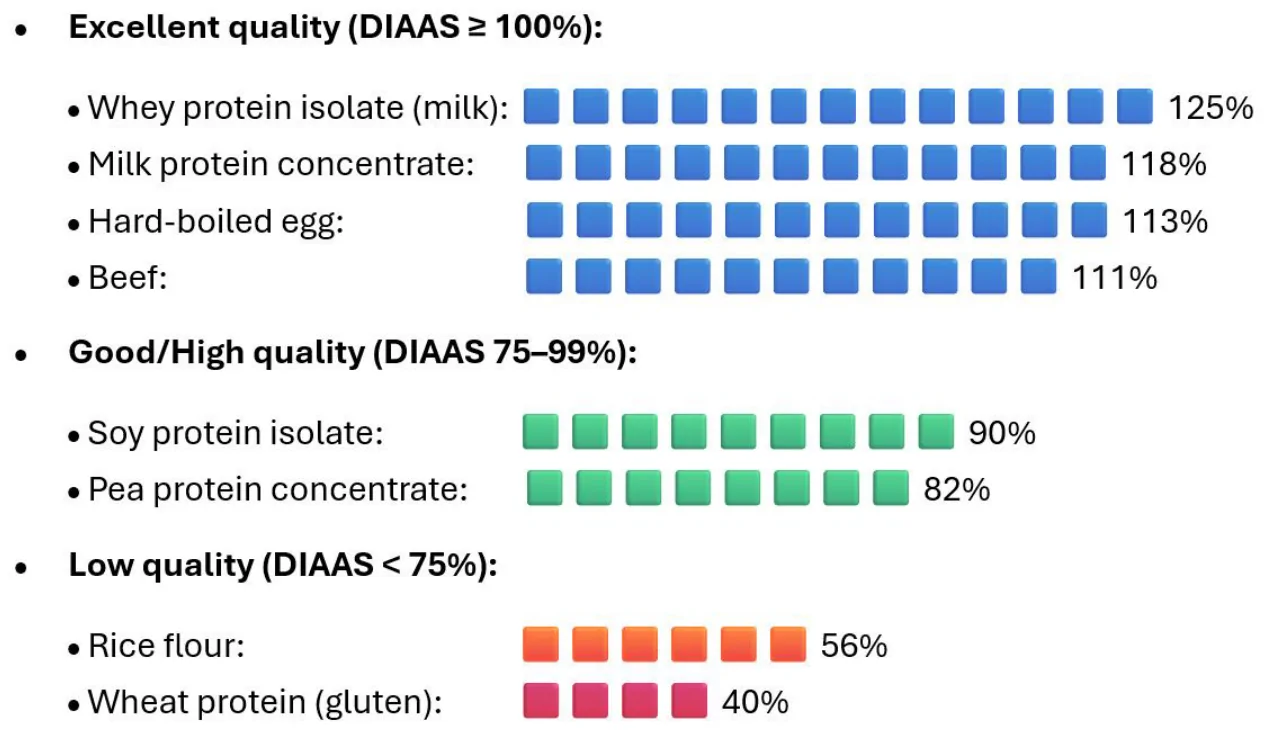
Comparative DIAAS values (%) for selected protein sources [10,14].

In 2013, FAO introduced the rigorous DIAAS (*Digestible Indispensable Amino Acid Score*). This methodology assesses protein quality by shifting the digestibility measurement point to the terminal ileum. This allows for precise determination of the actual amount of individual indispensableamino acids effectively absorbed in the small intestine. Furthermore, the DIAAS index is not subject to truncation at 100%, allowing for a more accurate representation of protein quality in superior sources (such as whey isolate, which reaches 125%) [10,14]. Comparative DIAAS values (%) for selected protein sources are shown in Figure 2.

Analysis of the distribution of values in Figure 2 indicates a clear division of the population of studied sources into three main clusters:

1. High cluster (DIAAS > 100%)—proteins of animal origin. Animal products (including milk protein isolate 118–143%, eggs 113%, beef 111%, chicken breast 108%) form a coherent group at the top of the distribution. Scores exceeding 100% indicate that these proteins not only provide all essential amino acids in proportions ideally suited to human needs, but also demonstrate very high pseudo-intestinal digestibility (true ileal digestibility). According to the FAO classification, they constitute a group of “excellent quality” proteins.
2. Medium cluster (DIAAS 75–99%)—plant isolates and concentrates, and selected legumes. Processed plant proteins (e.g., soy protein isolate ~90%, pea protein concentrate ~82–91%) and some traditional soy products (e.g., tofu, tempeh) fall within the range defined by the FAO as “good/high quality.” Refining processes remove dietary fiber and antinutritional factors, which increases their digestibility, but the presence of limiting amino acids (e.g., methionine and cysteine) prevents them from achieving results above 100%.
3. Distributed low distribution (DIAAS < 75%)—unprocessed plant products and cereals. The greatest range of values occurs in the group of whole plant products. The values range from moderate for legumes (e.g., lentils ~65–82%, cooked chickpeas ~83%), through lower for cereals and nuts (cooked rice ~59%, cooked wheat ~40%, almonds ~40%), to extremely low values for some cereals (e.g., corn products ~10%). The low level of the index in this group results from two factors: a strong deficit of key amino acids (e.g., lysine in cereals) and lower digestibility resulting from the presence of the natural plant matrix (fiber, phytates).

### Physiological and Molecular Determinants of the DIAAS Index

The profound disparity in DIAAS values observed between animal and plant proteins stems from their three-dimensional structure. The animal protein matrix facilitates direct access of proteolytic enzymes to peptide bonds:

- Whey and casein (dairy): Whey proteins are highly soluble globular proteins that undergo rapid gastric emptying, allowing their amino acids to be rapidly absorbed in the jejunum. Casein, in turn, forms a stable micellar coagulum under the influence of the acidic pH of the stomach. This slows its intestinal transit (“slow” protein), ensuring a steady, sustained release and absorption of amino acids, minimizing their premature hepatic deamination [10].
- Muscle and egg proteins: They have a unique fibrous structure (meat) or an excellent amino acid profile (ovalbumin). Their intestinal digestibility after thermal coagulationexceeds 95–97%.

In contrast, plant globulins (e.g., glycinin and β-conglycinin found in soy) are proteins with an extremely compact, oligomeric globular structure. This specific spatial conformation makes them exceptionally resistant to the action of pepsin in the acidic environment of the stomach and trypsin in the duodenum. Even aggressive hydrothermal treatment is unable to fully unfold these structures, drastically limiting the kinetics of their enzymatic degradation.

An additional factor reducing the DIAAS coefficient of raw plant materials ischemical modifications occurring during intensive industrial processing (e.g., the Maillard reaction, which irreversibly eliminates lysine from the available pool in the processes of creating isolates). Analyzing the amino acid profile reveals a profound deficiency of limiting amino acids in plant sources (lysine in cereals, sulfur amino acids in legumes) [10,13].

This means that when a diet is basedsolely on wheat gluten, the body is only able to utilize 40% of the supplied nitrogen for the synthesis of its own structural proteins, while the remaining 60% is inevitably oxidized in the urea cycle. This phenomenon implies the need for so-called protein complementation (e.g., simultaneous consumption of cereals and legumes) [2,9].

## 5. Absorption of Minerals: Iron and Zinc

### 5.1. Heme vs. Non-Heme Iron: Molecular Mechanisms and Dietary Interactions

**Figure 3:**
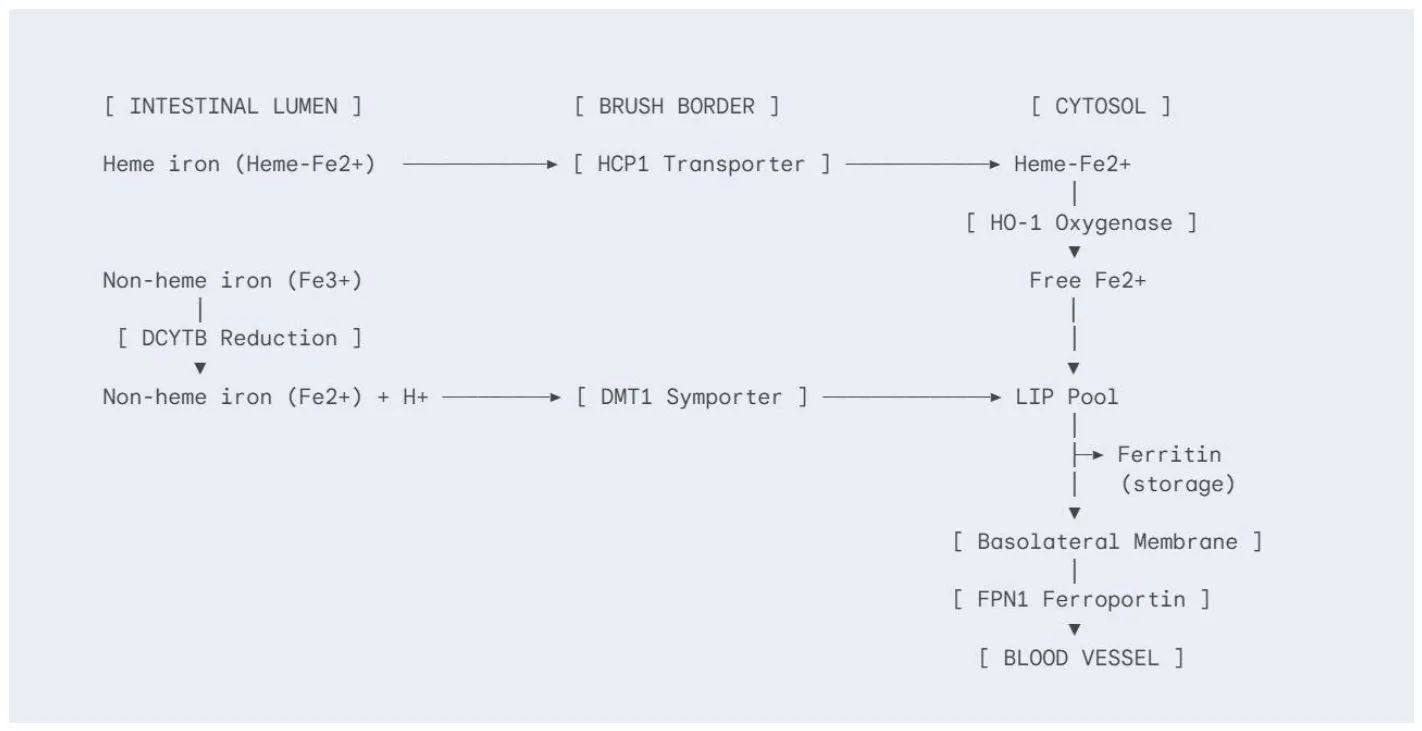
Simplified molecular topography of iron absorption in the duodenal enterocyte [1,3].

Iron, a key trace element involved in oxygen transport, ATP synthesis, and xenobiotic metabolism, occurs in food in two fundamentally different physicochemical forms. These forms determine the different kinetics, efficiency, and pathways of membrane transport across the duodenal enterocyte barrier [1,3,57,58,59,60,61,62]:

1. Heme iron (divalent Fe^2+^ cation coordinated within a porphyrin ring): This form is found exclusively in food products of animal origin, constituting an integral component of hemoglobin and myoglobin present in muscle tissues (red meat, offal, poultry, fish). The digestion of heme iron begins in the stomach, where, under the influence of pepsin and hydrochloric acid, the heme complex is proteolytically released from the globin protein shell. This released free heme remains highly soluble in the slightly alkaline environment of the small intestine, which prevents its polymerization. It is absorbed intact through active transport mediated by a specific enterocyte brush border transporter—HCP1 (Heme Carrier Protein 1). Once the heme complex enters the enterocyte cytosol, the porphyrin ring is enzymatically cleaved by the inducible enzyme heme oxygenase-1 (HO-1). This process releases the free Fe^2+^ cation, which supplies the intracellular labile iron pool (LIP). Because the iron cation is shielded by an organic ring until it crosses the cell membrane, heme iron is characterized by high resistance to the presence of chemical ligands in the chyme and the pH of intestinal juice. The average bioavailability of heme iron is high, ranging from 15% to as much as 35% [3].
2. Non-heme iron (free Fe^3+^ or Fe^2+^ ions and their inorganic salts): This is the only form of iron present in plant-derived foods (cereals, legumes, leafy vegetables) and the predominant form in synthetically enriched (fortified) foods and dietary supplements. The absorption of non-heme iron is a highly inefficient and physiologically complex process. Most non-heme iron compounds in the diet are present in the trivalent oxidation state (Fe^3+^), which, at the physiological pH of the duodenum, rapidly hydrates and precipitates as insoluble ferric hydroxide [1,3]. For absorption to be possible, Fe^3+^ ions must first be reduced to the soluble divalent form (Fe^2+^). This process is catalyzed by duodenal cytochrome b reductase (DCYTB), located on the apical membrane of the enterocyte, which uses intracellular ascorbate as an electron donor. Only Fe^2+^ ions can be transported into the cell via the DMT1 protein (Divalent Metal Transporter 1), a symporter coupled to the proton gradient (H^+^). Therefore, DMT1 activity is strictly dependent on the acidic microenvironment on the brush border surface. Any conditions associated with reduced gastric and intestinal acidity (e.g., the use of proton pump inhibitors—PPIs, or advanced age) drastically reduce the efficiency of this transport. The average bioavailability of non-heme iron is low, ranging from 2 to 10% [3]. The strategic selection of appropriate plant foods (legumes, whole grains, fruits, and vegetables) can significantly alter the dietary components that enhance or inhibit the intestinal solubility and absorption of non-heme iron [62,63,64]. A simplified molecular topography of iron absorption in duodenal enterocytes is shown in Figure 3.

The bioavailability of non-heme iron is subject to strong interactions with other dietary components that may act as enhancers or inhibitors of this process:

- Absorption enhancers: The strongest known activator of non-heme iron absorption is ascorbic acid (vitamin C). It works in two ways: it reduces Fe^3+^ ions to soluble Fe^2+^ in the digestive juice and forms stable, soluble chelates (iron ascorbate), preventing its precipitation during the passage of food into the distal sections of the small intestine [3,64,65,66].
- The “meat factor”: Another important enhancing factor is the consumption of meat (especially red meat, poultry, fish) along with sources of non-heme iron significantly increases the bioavailability of the latter. This mechanism is associated with the release of specific, low-molecular-weight polypeptides rich in sulfur amino acid residues (mainly cysteine) during meat digestion. These peptides demonstrate the ability to chelate free iron ions Fe^2+^, maintaining them in a soluble form and protecting them from reaction with inhibitors [8].
- Absorption inhibitors: The strongest blockers of non-heme iron bioavailability include phytates, polyphenols (including tannins found in tea, coffee, cocoa, and red wine), and dietary fiber. Phytic acid salts, which have a strong negative charge, form stable, high-molecular-weight, and insoluble complexes with iron cations (Fe^2+^ and Fe^3+^) at the physiological pH of the small intestine, completely preventing their interaction with the DMT1 transport protein [1,8,15].
- Calcium (Ca^2+^): This represents a unique inhibitor that has the ability to limit the absorption of both non-heme and heme iron [67,68,69]. Studies suggest that calcium competes for binding sites during intracellular iron transport or exerts a direct inhibitory effect on ferroportin (FPN1) expression on the basolateral membrane of the enterocyte, which prevents iron transfer to the portal circulation [5,6].

Many authors [70,71,72,73,74,75,76,77,78,79,80,81,82,83,84] report that vegetarians, especially vegetarian women, usually have lower serum ferritin levels.

#### Modern Forms of Iron in Food Fortification: NaFeEDTA (Sodium Ferric Edetate) and Iron(II) Bisglycinate

Although iron(II) sulfate has traditionally been a benchmark in bioavailability studies, its use in food fortification has significant limitations. Iron sulfate readily reacts with antinutritional substances (phytates, polyphenols) and can cause undesirable sensory changes, such as a metallic aftertaste or fat oxidation in the product. Therefore, advanced chelated forms, such as sodium ferric ethylenediaminetetraacetic acid (NaFeEDTA) and iron(II) bisglycinate, are increasingly being used in modern nutritional interventions and new-generation supplements.

Sodium ferric edetate. NaFeEDTA is an innovative compound with unique application properties, strongly recommended for the fortification of cereal and flour products, as reflected in World Health Organization guidelines. In the acidic environment of the stomach, the EDTA molecule tightly binds iron ions, protecting them from forming insoluble complexes with phytic acid, which is common in groats, bread, and legumes. As it passes into the duodenum, where absorption is most intense, iron is released from the complex and absorbed. Studies show that in meals high in phytate, the bioavailability of iron from NaFeEDTA is two to three times higher compared to classic iron(II) sulfate [85,86]. This makes this fortificant a highly effective tool for populations whose diet is based on unprocessed plant materials.

Iron(II) bisglycinate (amino acid chelate). Iron(II) bisglycinate is a structure in which one divalent iron ion (Fe^2+^) is bilaterally bonded to two molecules of the amino acid glycine. The resulting stable ring is electrically neutral and highly resistant to chemical agents in the gastrointestinal tract. This means that iron in this form does not react with absorption inhibitors (oxalates, tannins, or phytates) and bypasses the problem of competition with other minerals for transport pathways [87]. The bioavailability of bisglycinate has been shown to be 2.5 to over 3 times higher than that of conventional iron(II) sulfate, allowing for therapeutic effect with lower doses of elemental iron. Clinical tolerability is equally important—because free iron ions are not prematurely released in the stomach and do not irritate the mucosa, bisglycinate drastically reduces the occurrence of gastrointestinal symptoms (nausea, abdominal pain, constipation), which are the most common reason for patients to discontinue supplementation [88]. Both NaFeEDTA and iron(II) bisglycinate effectively bypass the biological barrier posed by the plant food matrix, rich in antinutritional substances, making modern fortified foods a much more reliable source of this element.

### 5.2. Zinc: Molecular Mechanisms of Absorption, Biochemistry of Phytate Inhibition, and Technological Strategies

Zinc (Zn^2+^) is a fundamental trace element in human physiology, being a catalytic and structural cofactor for over 300 enzymes and a key component of DNA-binding structural motifs, called zinc fingers, that control gene transcription [7,16]. Despite its abundance in the biosphere, the extent of zinc absorption in the duodenum and jejunum is highly variable and directly determined by the architecture of the food matrix and the presence of organic ligands.

Zinc absorption efficiency from animal-based foods, such as red meat, offal, fish, and crustaceans, is remarkably high, ranging from 30% to 40%. This is due to the abundant supply of basic and sulfur-containing amino acids (primarily histidine and cysteine) from animal tissues. These amino acids, released during proteolysis, act as highly effective, low-molecular-weight organic ligands that coordinate free Zn^2+^ ions. The resulting stable, neutral, and highly soluble chelates protect zinc from precipitation in the alkaline environment of intestinal juice, facilitating its presentation to the apical enterocyte transporter ZIP4 (encoded by the SLC39A4 gene). After passing through the apical membrane, zinc ions are buffered in the cytosol by metallothioneins (MT) or directly exported to the portal circulation by the basolateral transporter ZnT1 (SLC30A1) [7,16].

In contrast to animal-derived sources, zinc bioavailability from unprocessed plant foods (legumes, whole grains, nuts) is lower, reaching only 10–15%. The main biochemical factor responsible for this disproportion is phytic acid (myo-inositol-1,2,3,4,5,6-hexakisphosphate). At the physiological pH of the small intestinal lumen (≈6–7), the phosphate groups of phytate are almost completely deprotonated, acquiring a strong negative electrostatic charge. Zinc ions (Zn^2+^) exhibit extremely high coordination affinity for these ionized groups. This interaction leads to the formation of high-molecular-weight, insoluble and chemically inert zinc-phytate coordination network polymers, which are completely unavailable because they cannot interact with the binding domains of the ZIP4 transporter [7,8,15].

In order to quantitatively assess and predict the risk of subclinical zinc deficiency in populations following vegetarian, vegan, or cereal monoculture diets, nutritional medicine uses the molar phytate:zinc ratio, calculated according to the following Formula (1):

(1) $$\mathrm{phytate}:\mathrm{zinc}\;\mathrm{molar}\;\mathrm{ratio}=\;\frac{\mathrm{mmol}\;\mathrm{phytate}/\mathrm{day}}{\mathrm{mmol}\;\mathrm{zinc}/\mathrm{day}}=\;\frac{\mathrm{phytates}\;\left(\mathrm{mg}/\mathrm{day}\right)/660}{\mathrm{zinc}\;\left(\mathrm{mg}/\mathrm{day}\right)/65.4}$$

The World Health Organization (WHO) classifies diets based on this parameter, setting the limits of actual zinc absorption [7,8]:

- Molar ratio < 5 (high bioavailability): Typical of diets high in animal protein and low in unrefined fiber. Actual zinc absorption is then approximately 50–55%.
- Molar ratio 5–15 (moderate bioavailability): Characteristic of a varied diet containing a moderate proportion of grains and regular sources of animal protein. Actual absorption is approximately 30–35%.
- Molar ratio > 15 (low bioavailability): Common in strictly plant-based diets (vegetarian and vegan), where this index regularly reaches values of 20–35%. Zinc absorption then drops below 15%, which is a direct etiological factor of “hidden hunger” [7,8,11].

Zinc inhibition by phytate is further enhanced in the presence of a high calcium (Ca^2+^) supply. Calcium ions co-precipitate zinc, forming ternary, extremely insoluble calcium-zinc-phytate complexes, which almost completely block the paracellular and transcellular absorption pathways. Similar antagonism occurs at an Fe:Zn ratio exceeding 2:1 (e.g., with simultaneous supplementation with inorganic iron sulfate), where iron ions competitively block nonspecific zinc transport [8,15].

Chronic zinc deficiency caused by low bioavailability leads to serious clinical consequences:

1. Hypogeusia and dysgeusia: Impairments and anomalies of the sense of taste caused by dysfunction of gustin—a zinc-dependent metalloprotein of saliva responsible for the trophism of taste buds.
2. Profound Immunosenescence: Zinc is an essential cofactor thymic hormone—thymulin. Its deficiency prevents thymulin activation, leading to thymus involution, impaired T lymphopoiesis, and a drastic decline in cellular immunity.
3. Dermatological disorders and delayed wound healing: Resulting from the decrease in the activity of DNA polymerases and zinc-dependent matrix metalloproteinases (MMPs), key for keratinocyte proliferation and connective tissue remodeling.

To neutralize these unfavorable phenomena without having to abandon a plant-based diet, modern food technology and culinary arts implement methods aimed at enzymatic degradation of phytates by endogenous or exogenous phytases (myo-inositol hexakisphosphate phosphohydrolases). These enzymes hydrolyze phosphate ester bonds, releasing free inositol and orthophosphates, which permanently eliminates phytic acid’s ability to chelate zinc. Optimal kinetic conditions for these enzymes (pH 4.5–5.5 and temperature 45–55 °C) are achieved by [8,15]:

- Lactic acid fermentation: Carried out by lactic acid bacteria (LAB), which by lowering the pH activates both endogenous plant phytases and phytases synthesized de novo by microorganisms, reducing the level of phytates in cereal products by up to 90% [15].
- Soaking and germination: By hydrating the seeds, it abolishes their metabolic dormancy, inducing rapid synthesis of plant phytases and almost doubling the zinc bioavailability factor [8,15].

## 6. Calcium and Vitamin D: The Role of the Bioactive Dairy Matrix and Plant Antinutritional Ligands

Calcium (Ca^2+^) is a fundamental building block of the skeletal system and a critical secondary messenger controlling myocyte contractility, synaptic transmission, and blood coagulation cascades. The efficiency of its absorption in the gastrointestinal tract is determined not only by its absolute dietary concentration but primarily by the physicochemical structure of the surrounding food matrix [5,6].

Calcium from milk and dairy products is characterized by exceptionally high and stable bioavailability of 30–35%. This preferential absorption is a direct result of the synergistic action of a professional bioactive dairy matrix. Casein phosphopeptides (CPP—Casein Phosphopeptides) play an active role in it, which are specific oligopeptides released in the lumen of the duodenum and jejunum by controlled proteolysis of casein under the influence of pepsin and pancreatic enzymes (trypsin) [6].

These highly polar, negatively charged microdomains have the ability to form soluble, stable coordination complexes with divalent calcium cations (Ca^2+^). As chyme moves into the ileum, where pH increases to weakly alkaline values (≈7.2–7.5), free calcium ions naturally tend to precipitate as insoluble, amorphous calcium phosphate, Ca_3_(PO_4_)_2_.

Calcium binding by CPP effectively prevents this precipitation, maintaining calcium in a soluble and highly ionized form. This ensures a constant, high concentration gradient directly at the small intestinal mucosa, which drastically increases the efficiency of its passive, paracellular transport through the tight junctions of enterocytes (dependent on claudins 2 and 12) [6].

In plant-based foods, calcium bioavailability exhibits substantial variability, driven primarily by the concentration of oxalates and phytates [6]:

- Spinach, chard, sorrel: Despite the relatively high total calcium content, these products are an ineffective source of this element, as its bioavailability is estimated at less than 5%. This is due to the abundant presence of oxalic acid (H_2_C_2_O_4_). The acid associates with calcium, forming extremely insoluble calcium oxalate (CaC_2_O_4_), which is excreted from the body in the form of sharp mineral needles (raphides) [6]. The consequences of consuming oxalate-rich plant foods with a low dietary calcium intake are serious. Free oxalic acid, which has not been bound to calcium in the intestinal lumen, is absorbed into the portal circulation and excreted via the kidneys. In the renal tubules it combines with endogenous calcium, crystallizing in the form of kidney stones, which is the direct cause of calcium oxalate lithiasis (nephrolithiasis) and oxalate nephropathy [5].
- Cruciferous vegetables (*Brassica*—kale, broccoli, pak choi, mustard leaves): These products are characterized by minimal concentration of oxalic acid, thanks to which the calcium bioavailability index in them is record high and reaches 40–60%, exceeding the parameters of milk. Although calcium density is lower in plants, the bioavailability of calcium from low-oxalate green vegetables is also excellent. Some studies have also shown that net absorption from these sources may be higher due to the absence of casein-phosphopeptide complexes, which can sometimes inhibit absorption in sensitive individuals.

The regulation of calcium absorption at the systemic level is subject to strict hormonal supervision, in which the key element is the active form of vitamin D (1,25-dihydroxycholecalciferol, calcitriol). Calcitriol acts on the VDR nuclear receptor in duodenal enterocytes, inducing the expression of proteins responsible for active, saturable transcellular calcium transport. This process involves [6]:

1. Activation of the apical TRPV6 calcium channel.
2. Synthesis of the intracellular transport protein calbindin-D9k, which safely transports Ca^2+^ ions through the cell cytosol, preventing disturbances in intracellular calcium transmission.
3. Stimulation of the basolateral calcium pump PMCA1b (calcium-dependent ATPase), which exports ions into the bloodstream [6].

The efficiency of this process depends on the status and source of vitamin D in the body [6]:

- Vitamin D_3_ (cholecalciferol): Of animal origin (skin synthesis under UVB influence, oily marine fish, eggs). It exhibits high affinity for vitamin D-binding protein (DBP) in plasma. It is characterized by a long half-life and is a highly effective substrate for hepatic 25-hydroxylase (CYP2R1) and renal 1α-hydroxylase (CYP27B1).
- Vitamin D_2_ (ergocalciferol): Of plant and fungal origin (UV-irradiated ergosterol). It has significantly lower biochemical stability. D_2_ has a weaker affinity for DBP, resulting in its faster renal clearance and elimination from the body. Furthermore, ergocalciferol is more rapidly deactivated by the enzyme 24-hydroxylase (CYP24A1). Clinical studies show that vitamin D_2_ supplementation is 30–50% less effective in long-term increasing and maintaining plasma concentrations of the active metabolite 25(OH)D compared to vitamin D_3_, which further impairs calcium homeostasis in individuals on restrictive plant-based diets.

Some scientific reports emphasize that in individuals subjected to adequate, regular exposure to solar radiation (UVB), the origin and chemical form of vitamin D supplied through food play a secondary role [89]. Endogenous synthesis in the skin remains the physiologically most efficient mechanism for cholecalciferol production. It is estimated that short, moderate exposure of exposed skin to sunlight during the summer (even 10–15 min during midday hours) can generate doses of vitamin D_3_ significantly exceeding standard dietary recommendations [90].

Under conditions favoring effective cutaneous synthesis—taking into account the appropriate season, latitude, and the absence of physical and chemical barriers (UV filters)—the degree of vitamin D absorption from fortified foods or plant products is not critical for maintaining normal serum 25(OH)D concentrations. However, the absolute importance of dietary sources and supplementation becomes apparent under conditions of insufficient insolation—including in the case of: in the autumn-winter season at higher latitudes, in people spending most of their time indoors, in the elderly population or in people with a high skin phototype [91]. Taking skin synthesis into account allows for a more balanced and flexible approach to dietary recommendations.

## 7. Vitamin A and Vitamin B_12_

### 7.1. Physiology of Absorption and Molecular Mechanisms of Bioconversion of Provitamin A to Retinol

Vitamin A, defined as a group of fat-soluble organic compounds with the biological activity of all -trans -retinol, occurs in the diet in two diametrically different chemical forms: as ready-made, active vitamin A (preformed vitamin A) and as its plant precursors (provitamins A) [17,18].

In animal products (e.g., beef and pork liver, milk fats, egg yolk), vitamin A occurs mainly in the form of long-chain retinyl esters (mainly palmitate). The process of their assimilation in the duodenum begins with enzymatic hydrolysis catalyzed by pancreatic lipase, pancreatic cholesterol ester hydrolase, and mucosal brush border retinyl ester hydrolase. The released free retinol, exhibiting outstanding hydrophobicity, undergoes immediate solubilization inside mixed micelles (formed with the participation of bile acids, cholesterol and phospholipids), from where it penetrates the enterocyte membrane by facilitated diffusion with an efficiency of 70–90% [17,18].

Provitamin A, in turn, consists of plant isoprenoid pigments—carotenoids (primarily β-carotene, α-carotene, and β-cryptoxanthin). They are deposited within plant cell structures (chromoplasts), where they are strongly associated with membrane proteins and the pectin-cellulose matrix. The first critical step determining their bioavailability is the mechanical and thermal disintegration of cell walls (e.g., through blending, cooking). Carotenoids released from the matrix must be incorporated into mixed micelles. This process is extremely dependent on the co-presentation of lipids in the food. The minimum threshold of fat content in a meal necessary to initiate effective carotenoid micellization is approximately 3–5 g. In the absence of an adequate supply of lipids, hydrophobic carotenoids are unable to come into contact with the brush border of enterocytes and are excreted in the feces [17].

After entering the enterocyte, provitamin carotenoids undergo bioconversion to the active form of vitamin A. A key element of this pathway is the cytoplasmic metalloprotein enzyme β-carotene 15,15′-dioxygenase (BCO1). BCO1 catalyzes the symmetric cleavage of the central double bond (C_15_–C_15_′) of the β-carotene molecule with the participation of molecular oxygen, which theoretically should lead to the formation of two retinal (retinaldehyde) molecules. Retinal is then rapidly reduced to retinol by NADH/NADPH-dependent retinal reductase [17].

The efficiency of bioconversion of plant provitamins to active retinol varies dramatically depending on the dietary source and its degree of processing. Therefore, traditional retinol equivalents (RE) have been replaced by more precise retinol activity equivalent (RAE) measures [17]:

- 1 µg RAE = 1 µg of pure, crystalline retinol,
- 1 µg RAE = 2 µg retinol dissolved in oil (as a dietary supplement),
- 1 µg RAE = 12 µg dietary β-carotene provided by cooked, chopped vegetables,
- 1 µg RAE = 24 µg of other provitamin carotenoids (α-carotene, β-cryptoxanthin) contained in the plant matrix.

It should be emphasized that the 12:1 ratio for β-carotene is an average optimistic value. In the case of consumption of raw, whole vegetables (e.g., raw carrots), due to the barrier of intact cell walls, the actual bioconversion ratio may deteriorate, reaching values of 28:1 or even 36:1, which means that the actual absorption of provitamin A from a raw plant diet may be lower [17].

Another key barrier limiting the bioavailability of provitamin A is the host’s genetic conditions. Single nucleotide polymorphisms (SNPs) within the BCO1 gene determine the catalytic activity of this enzyme. In individuals genetically predisposed to the status of “poor converters”, the ability to convert plant β-carotene to retinol is drastically reduced (efficiency decrease by up to 60–80%) [18].

Another consequence in these individuals, despite a high intake of carotenoids in a vegan or vegetarian diet, is the risk of developing symptoms of vitamin A deficiency (e.g., impaired visual adaptation to darkness, so-called night blindness, xerophthalmia, hyperkeratosis) with the simultaneous occurrence of carotenoderma (orange discoloration of the skin of the hands and feet caused by the activation and accumulation of inefficiently metabolized β-carotene in the adipose tissue and the stratum corneum of the epidermis) [17,18]. This phenomenon proves that a plant-based diet rich in provitamins does not guarantee the satisfaction of the body’s metabolic needs for vitamin A without taking into account the genetic profile and appropriate modification of fat supply.

### 7.2. The Problem of Cobalamin (Vitamin B_12_) in Plant-Based Diets: Molecular Mechanisms of Absorption, the Problem of Analogues and Clinical Implications

Vitamin B_12_ (cobalamin) is the most structurally complex organometallic compound of key metabolic importance for the human body. A unique feature of cobalamin is the presence of a cobalt (Co) atom coordinated at the center of a macrocyclic corrin ring. Depending on the ligand attached to the cobalt atom, one can distinguish coenzymatically active forms (methylcobalamin participating in the methylation cycle homocysteine—methionine and adenosylcobalamin which is a cofactor for methylmalonyl-CoA mutase) and stable pharmaceutical forms (cyanocobalamin, hydroxocobalamin) [12,19]. Cobalamin is synthesized via a complex, multi-stage enzymatic pathway exclusively by specialized microorganisms (bacteria and archaea). Animals accumulate this vitamin in their tissues (especially in the liver, kidneys, and muscle tissue) as a result of symbiosis with the bacterial flora of the digestive tract (e.g., ruminants) or absorption from the trophic chain. Higher plants are completely devoid of the genes necessary for cobalamin synthesis, and their tissue matrix does not demonstrate a metabolic demand for this compound, which is why unprocessed food of plant origin does not contain the active form of vitamin B_12_ [12,14].

Cobalamin assimilation from food in humans is a highly inefficient and complex process, strongly dependent on the physiological integrity of the entire gastrointestinal tract. This process consists of several critical, sequential steps [4,12]:

- The gastric stage and the role of haptocorrin: After ingestion of animal food, cobalamin bound to matrix proteins is released by hydrochloric acid (HCl) and pepsin in the stomach. Immediately after release, free cobalamin is bound to haptocorrin (transcobalamin I, an R protein secreted abundantly by the salivary and gastric glands). This strong binding to haptocorrin in a highly acidic environment protects cobalamin from premature hydrolysis and acid degradation.
- Duodenal stage and the role of intrinsic factor (IF): After the contents enter the duodenum, alkaline pancreatic secretions and proteolytic enzymes (trypsin, chymotrypsin) digest the protein shell of haptocorrin, releasing free cobalamin. At the same time, it binds to a specific glycoprotein, intrinsic factor (IF). (IF, also known as Castle’s factor), produced by the parietal cells of the body and fundus of the stomach. The B_12_-IF complex is characterized by high resistance to enzymatic digestion and migrates unchanged to the final section of the small intestine.
- Ileal stage and receptor absorption: In the distal ileum, specific brush border membrane receptors—cubilin (part of the cubilin-amnionless receptor complex)—recognize and bind the B_12_-IF complex in the presence of calcium ions (Ca^2+^). The entire complex is endocytosed into the enterocyte. After enzymatic degradation of the complex in the cell’s lysosomes, free cobalamin is exported across the basolateral membrane into the portal circulation via the MRP1 transporter, where it immediately binds to transcobalamin II (TC-II), forming holotranscobalamin—the only biologically active circulating form capable of entering peripheral tissues via TC-II receptors [12,19].

Any attempts to find alternative, plant-based sources of active cobalamin in products commonly recommended in popular science literature, such as marine algae (*Spirulina*, *Chlorella*, *Nori*) or traditional Asian fermented foods (tempeh), encounter a biochemical barrier in the form of vitamin B_12_ analogues (corrinoids/pseudovitamin B_12_). Pseudovitamins (such as adenine cobyramide) are characterized by an almost identical corin ring structure, but have a differently shaped nucleotide base attached to the axial cobalt moiety (e.g., adenine instead of 5,6-dimethylbenzimidazole). This subtle geometric modification renders B_12_ pseudovitamins completely devoid of metabolic activity in human cells [12,14].

Moreover, the presence of B_12_ pseudovitamin in the diet can have two highly harmful health consequences:

- Competitive blocking of absorption: Pseudovitamins exhibit very high affinity for human haptocorrin and cubilin receptors in the ileum. Consuming products rich in these analogues (e.g., spirulina) may results in competitive saturation of transporters and blocking the absorption of rare, trace amounts of real, active cobalamin, which may accelerate the development of profound systemic deficiencies [12,14].
- Laboratory diagnostic bias: Commonly used medical diagnostic tests for determining serum vitamin B_12_ levels (based on radioimmunoassay or chemiluminescence methods) lack sufficient specificity and do not distinguish active cobalamin from its inactive corrinoid analogues. As a result, vegans consuming algae may obtain false-positive laboratory test results (within the reference range), while at the cellular level they develop an extreme deficiency, leading to irreversible multi-organ damage [12,19]. The only reliable diagnostic method in these groups remains the determination of methylmalonic acid (MMA) levels in urine or serum and the level of active holotranscobalamin (holo-TC).

Chronic deficiency of active cobalamin caused by a restrictive plant-based diet without proper supplementation leads to the development of three interrelated pathophysiological syndromes with a dramatic clinical course [12,19]:

1. Megaloblastic anemia and the folate trap phenomenon: Cobalamin in the form of methylcobalamin is an essential cofactor of the folate synthase enzyme. methionine, which catalyzes the conversion of homocysteine to methionine. This reaction is accompanied by the simultaneous conversion of 5-methyltetrahydrofolate (5-MTHF) to tetrahydrofolate (THF). In the absence of vitamin B_12_, this cycle is completely blocked. Folates in the body are irreversibly “trapped” in the 5-MTHF form, which prevents their regeneration into the active coenzymatic form (e.g., 10-formyl-THF or 5,10-methylene-THF) necessary for the de novo synthesis of purines and monophosphate. thymidylate—a key building block of DNA [12]. Disturbance in nucleic acid synthesis primarily affects rapidly dividing red blood cell lines in the bone marrow. Asynchrony of nuclear-cytoplasmic maturation occurs—the cell nucleus cannot keep up with division, while hemoglobin synthesis continues uninterrupted in the cytoplasm. This results in the formation of giant, immature red blood cells—megaloblasts (macrocytosis, MCV > 100 fl)—susceptible to premature hemolysis, and severe megaloblastic anemia [12,19].
2. Subacute combined spinal cord degeneration (SCD—Subacute Combined Degeneration): This is the most severe, often irreversible neurological complication of B_12_ deficiency. Adenosylcobalamin is necessary for the proper functioning of the enzyme mutase Methylmalonyl-CoA, which converts methylmalonyl-CoA to succinyl-CoA (which enters the Krebs cycle) [19]. Blockade of this pathway leads to a pathological increase in the concentration of methylmalonic acid (MMA) and propionic acid in body fluids. These toxic metabolites are massively incorporated into the myelin sheaths of nerve fibers of the central and peripheral nervous system instead of normal fatty acids. Furthermore, the lack of methionine synthesis impairs the production of S-adenosyl-L-methionine (SAM)—a universal methyl donor necessary for the methylation of myelin basic protein (MBP) [19]. The consequence is rapid demyelination of the posterior cords (responsible for proprioception and vibration) and lateral cords (pyramidal tracts controlling motor function) of the spinal cord, as well as the peripheral nerves and optic nerve. Patients initially report symmetrical paresthesias (tingling, numbness in the hands and feet), which progress to sensory ataxia (ataxia, loss of balance, positive Romberg’s sign), spastic limb paresis, and in advanced stages—severe neurodevelopmental and neuropsychiatric disorders (so-called megaloblastic madness, dementia, depression) and vision loss [12,19].
3. Hyperhomocysteinemia and endothelial damage: Methylation blockade Homocysteine causes a rapid increase in its concentration in blood serum. Homocysteine exerts direct cytotoxic and prooxidant effects on vascular endothelial cells. It induces membrane lipid peroxidation, stimulates vascular smooth cell proliferation, and activates blood coagulation cascades by inhibiting thrombomodulin expression and factor V activation. Hyperhomocysteinemia is an independent, strong risk factor for the development of atherosclerosis, venous thrombosis, myocardial infarction, and ischemic stroke [19].

For this reason, from the point of view of modern evidence-based medicine (EBM), plant-based diets (especially vegan ones) absolutely require constant, controlled and professionally selected supplementation of active vitamin B_12_ (most often in the form of stable cyanocobalamin in doses of 50–250 µg per day or 2000 µg once a week, which allows for covering the demand by passive transport—simple diffusion occurring with an efficiency of only 1% outside the cubilin receptor mechanism) [2,12].

## 8. Implications for Contemporary Nutrition Strategies and Public Health Policy: Combating “Hidden Hunger”

Understanding the fundamental differences in nutrient density and the actual bioavailability of macro- and micronutrients is critical for designing sustainable food systems and optimizing global public health strategies. Dynamic demographic and environmental changes are prompting governments and international organizations to promote a dietary transition consisting of a reduction in the consumption of animal-based foods (ASF) in favor of diets based primarily on plant-based raw materials [2,9]. Although this reduction is justified from an ecological perspective (reduced greenhouse gas emissions, a smaller carbon footprint, and conservation of water and land resources), its uncritical popularization, lacking a thorough consideration of bioavailability, carries a direct health threat in the form of a dynamic return of the “hidden hunger” phenomenon [11]. This condition is characterized by a lack of dramatic, immediate clinical symptoms of protein-calorie malnutrition (such as kwashiorkor or marasmus), yet it causes insidious, long-term degradation of the population’s physical and mental health. These effects manifest most rapidly and dramatically in so-called vulnerable (physiologically sensitive) groups:

- Children and adolescents in the phase of intensive somatic growth: Insufficient supply and low bioavailability of zinc, iron and calcium lead to growth retardation, delayed psychomotor development, bone mineralization disorders and cognitive impairment (permanent decrease in IQ caused by iron deficiency at the stage of myelination of brain structures) [11].
- Women of reproductive age, pregnant and breastfeeding: Increased demand for iron (necessary for the construction of the placenta and fetal erythropoiesis), calcium and B vitamins creates an extreme risk of anemia, neural tube defects in the fetus (in case of folic acid and vitamin B_12_ deficiency) and perinatal complications, including low birth weight of newborns [11].
- Elderly (geriatric) individuals: In these individuals, the naturally occurring process of gastric mucosal involution, decreased secretion of hydrochloric acid and intrinsic factor (IF), limits the absorption of micronutrients. Compounding this with a restrictive plant-based diet with low nutrient density and low protein bioavailability (low DIAAS values) may accelerate the development of osteopenia, osteoporosis, and sarcopenia (loss of muscle mass and strength), leading to loss of motor autonomy and increased mortality [9].

To eliminate these critical disparities and prevent a global crisis of qualitative malnutrition, modern food technology, biotechnology, and agricultural engineering are undertaking coordinated efforts to optimize the bioavailability of micronutrients from the plant matrix. These strategies encompass three main areas of implementation [8,15]:

1. Artificial biofortification and industrial food fortification:
  ○ Industrial fortification (enrichment): This involves the physical addition of pure, standardized chemical compounds directly to commonly consumed plant matrices (e.g., wheat flour, corn flour, plant-based milk substitutes). Traditional fortification with cheap inorganic salts (e.g., iron sulfate, zinc oxide) encounters significant limitations, as these compounds exhibit low bioavailability and engage in negative sensory interactions. Therefore, modern technology implements advanced, organic, and chelated mineral carriers, such as iron bisglycinate or zinc gluconate and picolinate [8].
  ○ Agrochemical and genetic biofortification: This is the process of increasing the content and bioavailability of nutrients in crop plants during their growth stage. This is achieved through selective breeding, foliar fertilization with micronutrients, or advanced genetic engineering [8,17].
2. Molecular enzymatic innovations in food processing:
  ○ Phytase Applications: This involves the addition of exogenous of microbial phytases to flours, sourdough starters, and feeds during the mixing and hydration stages. These enzymes exhibit high thermal resistance, permanently breaking down phytic acid into lower inositol phosphates, which do not have zinc and iron chelating properties [15].
  ○ Enzymatic cell wall degradation: This involves the use of cellulase, hemicellulases, and xylanase complexes. These enzymes precisely degrade the stiff, lignocellulosic scaffolds of legume and cereal cell walls. This process allows for the physical release of intracellular plant globulins and albumins, exposing them directly to endogenous proteases in the human gastrointestinal tract [10,13].
3. Advanced and optimized culinary techniques at the household level: Food technology promotes the renaissance of traditional biochemical seed processing methods, optimizing their physicochemical parameters to maximize absorption [8,15]:
  ○ Controlled soaking process: Conducted at elevated temperature (45–55 °C) in a slightly acidic environment (through the addition of lemon juice). Such parameters activate dormant endogenous phytases present in cereal grains [15].
  ○ Germination: Initiates rapid metabolic processes in the seed, during which reserve proteins and minerals are mobilized. Natural synthesis of phytases and amylases occurs [8,15].
  ○ Lactic acid fermentation with the participation of selected LAB strains: It allows for a significant reduction in pH, which not only directly promotes the solubility of mineral ions, but also deactivates plant protease inhibitors [8].

### 8.1. Other Effective Dietary Strategies for Optimizing Micronutrient Absorption

The total content of minerals and vitamins in the diet does not equate to their bioavailability. An important element of dietary interventions is the use of proven dietary strategies that minimize the effects of antinutritional substances and utilize synergistic interactions between nutrients.

Synergy of Vitamin C and Non-Heme. Iron Iron found in plant-based foods (non-heme iron) is characterized by relatively low absorption (usually 2–20%) compared to heme iron from animal products. Concomitant intake of ascorbic acid (vitamin C) in a single meal is one of the most effective methods for increasing the absorption of this element [92]. This mechanism is based on the dual action of ascorbic acid: first, it reduces trivalent iron ions to much better absorbed divalent ions, and second, in the acidic environment of the stomach, it forms soluble chelates with iron, preventing its precipitation at the higher pH of the duodenum. In clinical practice, it is recommended to deliberately combine plant sources of iron (e.g., lentils, spinach, amaranth) with foods rich in vitamin C (e.g., red peppers, berries, citrus fruits, and parsley).

Phytate reduction through fermentation (sourdough bread). Phytic acid (phytates), commonly found in the outer coverings of cereals, legumes, nuts, and seeds, is a potent inhibitor of mineral absorption. It forms insoluble salts with iron, zinc, calcium, and magnesium in the gastrointestinal tract, preventing their absorption. An effective strategy for neutralizing phytates is the process of lactic acid fermentation, traditionally used in the production of sourdough bread. Acidification of the dough environment (a drop in pH) activates endogenous phytases—enzymes capable of dephosphorylating phytic acid—and provides enzymes produced by lactic acid bacteria. Research shows that extended sourdough fermentation can degrade most phytates (up to 60–70% more effectively than short yeast fermentation), drastically increasing the bioavailability of zinc, magnesium, and iron [93].

### 8.2. Bioavailability of Omega-3 Fatty Acids and the Role of Microalgae Oil

Another key challenge in fully plant-based diets is the adequate supply of long-chain polyunsaturated fatty acids (LC-PUFAs) from the omega-3 family, particularly eicosapentaenoic acid (EPA) and docosahexaenoic acid (DHA). Traditional vegan diets provide omega-3 essentially exclusively in the form of alpha-linolenic acid (ALA), found in foods such as flaxseed, chia seeds, rapeseed oil, and walnuts. Although the human body possesses enzymatic pathways (dependent on elongases and desaturases) capable of endogenously converting ALA to EPA and ultimately to DHA, the efficiency of this process is physiologically very low. It is estimated that in adults, the conversion of ALA to EPA typically ranges from 5% to 10%, and to DHA often does not exceed 0.5% to 1%, or is even difficult to detect [94]. For this reason, relying solely on plant sources of ALA may be insufficient to optimize the omega-3 index (the percentage of EPA and DHA in red blood cells), which is crucial for the cardiovascular and neurological systems. For individuals excluding animal-source foods (ASF), including fish and fish oils, the most effective dietary strategy is direct supplementation with pre-formed EPA and DHA obtained from microalgae cultures (e.g., *Schizochytrium* sp. or *Crypthecodinium cohnii* species). It is important to emphasize that microalgae are the primary source of omega-3 fatty acids in the marine food chain—fish and marine mammals merely accumulate them in their tissues. Interventional studies clearly demonstrate that microalgae oil is fully bioequivalent to traditional fish oil in terms of absorption and clinical efficacy [95]. Algae-based preparations allow for the direct delivery of LC-PUFAs, bypassing the inefficient conversion pathway, providing an ecologically sustainable solution free from ocean pollution (e.g., heavy metals) for patients on vegan diets.

## 9. Limitations of the Review Article and Comprehensive Analysis

Comprehensive analysis and synthesis of scientific data comparing the bioavailability of nutrients from different dietary sources encounters a number of specific methodological, biological, and technological limitations. Identifying these barriers is essential for a reliable interpretation of the presented results and for determining precise vectors for future empirical research:

1. Methodological heterogeneity and protocol standardization bias: A key methodological limitation encountered when aggregating data from Web of Science, PubMed, Scopus, and ScienceDirect is the lack of standardized analytical protocols for measuring bioavailability. In vitro studies (e.g., using static enzymatic reactor digestion models) provide valuable data on phase-specific bioavailability but do not replicate dynamic intestinal motility, the influence of neuroplexuses, and humoral hormonal regulation in humans [4]. Clinical trials, on the other hand, are characterized by extreme variability in the selection of assay methods.
2. Interindividual variability, epigenetic variation, and the gut metagenome: Reviews necessarily rely on statistical averages, which mask the powerful influence of the individual host’s metabolic profile. Bioavailability between individuals is highly personalized, determined by:
  ○ Genetic and epigenetic factors: Going far beyond the BCO1 gene polymorphism discussed in Section 7 [17,18].
  ○ Metagenome (microbiota): The composition of the bacterial flora of the small and large intestine demonstrates the ability to synthesize de novo some vitamins and to produce endogenous bacterial phytases, capable of partial hydrolysis of phytic acid.
3. Barriers to industrial scaling and the economics of technological innovation: Biotechnological innovations discussed here, such as food supplementation with recombinant phytases or enzymatic degradation of lignocellulosic structures of plant cell walls, demonstrate high efficiency under controlled laboratory conditions. However, their implementation on a mass industrial scale encounters significant engineering barriers (e.g., thermal instability of enzymes) [15].

### Methodological Limitations of Bioavailability Studies: The Role of the Food Matrix and the Reductionist Model

When interpreting data on micronutrient bioavailability, a key methodological limitation of the existing literature must be considered: a significant portion of experimental studies are based on a reductionist model. These studies commonly utilize single nutrient isolates (e.g., aqueous mineral solutions or synthetic vitamin capsules) analyzed in the laboratory or under fasting conditions, rather than evaluating whole foods consumed as part of complex, daily meals. This approach, while essential for identifying basic metabolic pathways and individual interactions (e.g., between iron and ascorbic acid), completely ignores the fundamental concept of the “food matrix.” In natural plant materials, vitamins and minerals do not occur in free form but are integrally incorporated into complex, three-dimensional physicochemical structures—such as intact cell walls, protein-lipid complexes, or dense dietary fiber networks. This matrix determines the rate of release (bioaccessibility) of nutrients during chewing and enzymatic digestion. Administering minerals in the form of isolates completely bypasses these natural release barriers, leading to a systematic overestimation of their actual absorption in humans [96].

Furthermore, studies using isolates do not reflect the dynamics of digestion of a complete meal. The daily diet is characterized by the simultaneous supply of hundreds of biologically active compounds that interact in multiple ways, often competitively. For example, while the addition of vitamin C to a simple iron solution can exponentially increase its absorption, the same supplement incorporated into a complex, multi-ingredient meal (rich in calcium, which competes for absorption, iron-binding phytates, and polyphenols) produces a much smaller stimulating effect [97]. Extrapolating results from isolated models to real populations consuming diverse, nutritious diets is therefore challenging and may lead to inaccuracies in constructing practical dietary recommendations, including those aimed at vegans.

## 10. Conclusions

In summary, a comprehensive bioavailability analysis reveals that the structural matrix of animal-derived foods exhibits evolutionarily optimized, unique, and highly coordinated properties, facilitating maximum absorption efficiency of key macronutrients and micronutrients in the human gastrointestinal tract. The exceptional nutrient density and bioavailability of proteins (expressed by the DIAAS index, free of fecal estimation error), heme iron (resistant to inhibitors and transported via a specific HCP1 pathway), zinc and calcium (stimulated by organic matrix cofactors such as CPP and specific amino acid ligands), and preformed vitamins (retinol with a high RAE coefficient, metabolically stable vitamin D_3,_ and exclusively animal-derived cobalamin) place these products in an unrivaled position in terms of deficiency prevention and support for anabolic processes.

While a fully balanced diet without animal-based ingredients is theoretically and practically feasible in terms of meeting energy needs, its safe implementation in the long term presents consumers and food technologists with complex physiological and biochemical barriers. The clinical success of plant-based models depends crucially on advanced knowledge of nutritional synergies and antagonisms, the elimination of plant antinutritional ligands (phytates, oxalates, protease inhibitors) through targeted processing techniques (LAB fermentation, germination, hydrothermal activation of endogenous phytases), and rigorous, targeted pharmacological supplementation of vitamin B_12_, essential to prevent irreversible demyelinating neuropathies and megaloblastic anemia.

Consequently, contemporary dietary recommendations and public health policies, striving to reduce the environmental footprint, must strictly take into account the biophysical phenomenon of the matrix effect and the genetic heterogeneity of the human population in terms of the efficiency of bioconversion pathways (e.g., BCO1 gene polymorphisms), in order to avoid a widespread public health regression caused by “hidden hunger”. It is also important that the best diet is both nutritionally adequate and sustainable for the individual and the environment.

## Figures and Tables

**Table 1:** Comparison of chemical forms, primary sources, and average bioavailability coefficients of essential nutrients in humans.

Nutrient	Main Sources (Animal/Plant)	Form in Animal Products	Average Bioavailability	Form in Plant Products	Average Bioavailability	Main Factors Determining the Difference	Characteristics of the Study Population	Experimental Conditions and the Effect of the Food Matrix *	References
Protein (quality)	Meat, fish, dairy, eggs/Legumes, cereals, nuts	Complete (full amino acid profile)	DIAAS ≥ 100%	Incomplete (limiting amino acids)	DIAAS < 90% (often <70%)	Presence of protease inhibitors, rigid cell wall.	Healthy adults and standardized animal models (cannulated pigs, rats) were used to determine DIAAS and PDCAAS indices for human requirements.	Measurements are primarily based on true ileal digestibility. Lower values result from protein entrapment in the intact fiber matrix and the presence of protease inhibitors (e.g., trypsin in undercooked soybeans).	[2,9,10,14]
Iron	Offal, red meat, blood/Spinach, legumes, cereals	Heme (Fe^2+^) in porphyrin)	15–35%	Non-heme (inorganic Fe^3+^)	2–10%	Phytates, polyphenols, ascorbic acid, “meat factor”.	Experimental Conditions and Effects of Food Matrix Healthy adults (both women and men). Noticeably higher absorption (closer to 20%) in individuals diagnosed with baseline iron deficiency (upregulated by hepcidin)	Isolated test meals administered under fasting conditions. Lower limits (2–5%) observed in meals high in phytates (e.g., bran, soy) and tannins; upper limits in the presence of high doses of vitamin C.	[1,3,8]
Zinc	Oysters, red meat, liver/Pumpkin seeds, legumes, cereals	Free cation coordinated by ligands	30–40%	Free cation strongly chelated	10–15%	Phytate:zinc molar ratio, co-precipitation with calcium.	Healthy adults on plant-based diets. The body does not exhibit such strong compensatory mechanisms (increased absorption) for zinc as it does in iron deficiency.	Studies using stable isotopes. Bioavailability is drastically reduced by a high molar ratio of phytate to zinc (phytate:zinc ratio > 15:1). Techniques such as soaking, sprouting, and lactic acid fermentation significantly improve this result.	[7,8,16,17]
Calcium	Milk, rennet cheeses, yogurt/Spinach, kale, broccoli	Ionized, soluble (CPP complexes)	30–35%	Bound, insoluble (oxalates)	<5% (spinach) to 50% (kale)	Presence of oxalic acid, low density in brassica vegetables.	Healthy adults with balanced calcium-phosphate status and normal serum vitamin D levels.	Meals devoid of oxalates. These values drop dramatically (to approximately 5%) in vegetables high in oxalic acid (e.g., spinach, beet greens) assessed under similar isotopic conditions.	[5,6]
Vitamin A	Liver, butter, cod liver oil, egg yolks/Carrots, pumpkin, sweet potatoes	Preformed retinol (retinyl esters)	70–90%	Provitamins (e.g., β-carotene)	Variable (RAE ratio 12–24:1)	Presence of dietary fats, BCO1 gene polymorphism.	Healthy individuals, taking into account the enormous individual variability. Approximately 30–50% of the population possess polymorphisms of the BCO1 gene (so-called “poor converters”), which genetically reduce the ability to convert provitamin A into its active form.	Extreme dependence on the food matrix. The lowest bioavailability (approximately 28:1) is found in raw, firm leafy vegetables. Disruption of the cell walls (blending, cooking) and the necessary addition of fat in the meal (which determines micelle formation) improve conversion (closer to 12:1).	[18,19]
Vitamin B_12_	Beef, offal, fish, dairy/No natural sources	Active cobalamin	High (IF-depende nt)	Inactive analogs (pseudovitamin B_12_)	0% (no activity in humans)	Lack of synthesis in plants, competitive receptor blockade.	Vegans and vegetarians, as well as elderly individuals whose gastric atrophy impairs B_12_ absorption from food (but not from crystalline supplements).	Absorption depends on the saturation of the carrier protein (Castle Factor) in the ileum—this process becomes saturated at a dose of approximately 1.5–2 µg per meal. Therefore, for high doses of 500–1000 µg, absorption relies almost exclusively on very inefficient passive diffusion (approximately 1% of the administered dose).	[12,15,20]
Omega-3 Fatty Acids	Wild salmon, mackerel, herring/Flaxseed, nuts	Active forms: EPA and DHA	High (direct assimilation)	Precursors: ALA (α-linolenic acid)	Low (conversion to EPA < 5%, DHA < 1%)	Inefficient enzymatic pathway, competition with Omega-6.	Primarily healthy young men. In women of reproductive age, the conversion rate is higher (up to approximately 21%) due to the stimulating effect of endogenous estrogens.	Studies using labeled stable isotopes (13C-ALA). Background diets characterized by a typical Western diet with a high intake of linoleic acid (LA), which competitively inhibits the enzyme Δ6-desaturase.	[21]

Explanations: * Much of the literature data is based on single-meal radioisotope studies. Therefore, they may not fully reflect long-term systemic adaptation (e.g., compensatory increase in mineral absorption from the gastrointestinal tract in response to prolonged low intake in a full-value vegan diet).

**Table 2:** Summary of bioavailability parameters of selected nutrients in clinical studies.

Nutrient/Study Parameter	Animal Matrix (ASF)	Clinical Bioavailability	Alternative Plant Matrix	Clinical Bioavailability	Conclusions and Clinical Implications	References
Protein (True ileal digestibility)	Chicken egg (cooked)	97% ± 1.2%	Pea concentrate	80% ± 2.8%	Weaker proteolysis of plant proteins results from structural cell wall barriers.	[4,10,14]
	Cow’s milk	95% ± 1.5%	Wheat gluten	71% ± 3.4%		
Iron (Net absorption percentage in humans)	Beef (heme)	25–30%	Spinach (non-heme)	1.4–3.0%	Non-heme absorption is extremely low, but increases with ascorbate intake.	[1,3]
	Fish (heme)	18–22%	Legumes + vit. C	12–15%		
Zinc (Fractional plasma absorption)	Red meat	45–55%	Whole grains, legumes	12–18%	High phytate ratios drastically limit zinc uptake.	[7,8,17]
Calcium (Fractional absorption)	Cow’s milk (CPP)	32.1% ± 1.5%	Spinach (oxalates)	5.1% ± 0.9%	Calcium from spinach crystallizes. Kale has higher bioavailability but low density.	[5,6]
			Kale	49.3% ± 2.1%		
Vitamin A (Provitamin bioconversion)	Retinyl esters	80–90%	β-carotene in raw carrot	3–5%	Conversion from vegetables to retinol is extremely inefficient without added dietary fat.	[18,19]
Vitamin B_12_ (Absorption at specific doses)	Low dietary doses	50–60%	High supplement doses	≈1%	At therapeutic doses, receptor-mediated absorption becomes saturated (passive diffusion).	[12,20]
Omega-3 (Synthesis of EPA/DHA from ALA)	Fatty fish (direct consumption of EPA/DHA)	95–98%	ALA (flaxseed)	ALA → EPA: 5–8%	Conversion of ALA to DHA and EPA is extremely low due to limited desaturase activity.	[21]
			Men (ALA → DHA)	ALA → DHA: <1%		

## Data Availability

No new data were created or analyzed in this study. Data sharing is not applicable to this article.

## Abbreviations

The following abbreviations are used in this manuscript:

AAS	Amino Acid Score
ALA	alpha-linolenic acid
ANFs	Factors anti-nutritional factors
ASF	Animal-Source Foods
BCO1	β-carotene dioxygenase 15,15′-monooxygenase
BCO2	β-carotene dioxygenase 9′,10′-monooxygenase
CPP	Casein Phosphopeptides
DBP	Vitamin D-Binding Protein
DCYTB	Reductase duodenal cytochrome B
DHA	Docosahexaenoic acid
DIAAS	Digestible Indispensable Amino Acid Score
DMT1	Metal transporter bivalent 1
EBM	Evidence-Based Medicine
EGCG	Gallate epigallocatechin
EPA	Eicosapentaenoic acid
HCP1	Protein transporting heme 1
IF	Intrinsic Factor
LAB	lactic acid bacteria
LAL	Lysinoalanine
LIP	Labile Iron Pool
MMA	Methylmalonic acid
MPS	Muscle Protein Synthesis
PDCAAS	Protein Digestibility-Corrected Amino Acid Score
RAE	Equivalent activity retinol
RCT	Randomized test controlled
SCD	Subacute connected degeneration core
SNP	Polymorphism single nucleotide
VDR	Vitamin D Receptor
WHO	World Health Organization
ZIP4	Zinc transporter ZIP4 (SLC39A4)
ZnT1	Zinc transporter ZnT1 (SLC30A1)
